# New perspectives in unresectable cholangiocarcinoma? Evaluation of chemosaturation with percutaneous hepatic perfusion as a palliative treatment option

**DOI:** 10.1007/s10585-022-10193-4

**Published:** 2022-11-22

**Authors:** Cornelia L. A. Dewald, Lena S. Becker, Timo C. Meine, Sabine K. Maschke, Frank K. Wacker, Anna Saborowski, Arndt Vogel, Jan B. Hinrichs

**Affiliations:** 1 Institute for Diagnostic and Interventional Radiology, Hannover Medical School, Carl-Neuberg-Straße 1, 30625 Hannover, Germany; 2 Department of Gastroenterology, Hepatology and Endocrinology, Hannover Medical School, Hannover, Germany

**Keywords:** Percutaneous hepatic perfusion (PHP), Cholangiocarcinoma (CCA), Melphalan, Intra-arterial therapy, Interventional oncology

## Abstract

Cholangiocarcinoma (CCA) are the second most common primary liver tumors and carry a dismal prognosis. Chemosaturation with percutaneous hepatic perfusion (PHP) is a palliative, intra-arterial therapeutic approach that provides a high dose chemotherapy of the liver with reduced systemic exposure. Aim of this retrospective, monocentric study was to analyze PHP as a palliative treatment for unresectable CCA. Toxicity, adverse events and complications were classified using the Common Terminology Criteria for Adverse Events (CTCAE v5.0). Overall response rate (ORR) and disease control rate (DCR) were evaluated according to the Response Evaluation Criteria in Solid Tumors (RECIST1.1). Median overall survival (mOS), median progression-free survival (mPFS) and hepatic mPFS (mhPFS) were computed using Kaplan–Meier estimation. In total 17 patients were treated with 42 PHP between 10/2014 and 09/2020. No significant complications occurred during the interventions. mOS was 27.6 (interquartile range (IQR) 16.5–37) months from first diagnosis and 9.9 (IQR 3.8–21) months from first PHP. mPFS was 4 (IQR 2–7) and mhPFS was 4 (IQR 3–10) months. ORR was 25% and DCR 75%. Significant, but transient hematotoxicity was frequent with grade 3/4 thrombopenia after 50%, leukopenia after 26% and anaemia after 21% of the interventions. An increase of transaminases (AST increase after 21% and ALT increase after 14% of the PHP) developed more often than a deterioration of the liver synthesis capacity. Salvage treatment with PHP has the potential to prolong life in selected patients with unresectable, refractory cholangiocarcinoma. The interventional procedure is safe. Post-interventional toxicity is frequent but manageable.

## Introduction

**Fig. 1:**
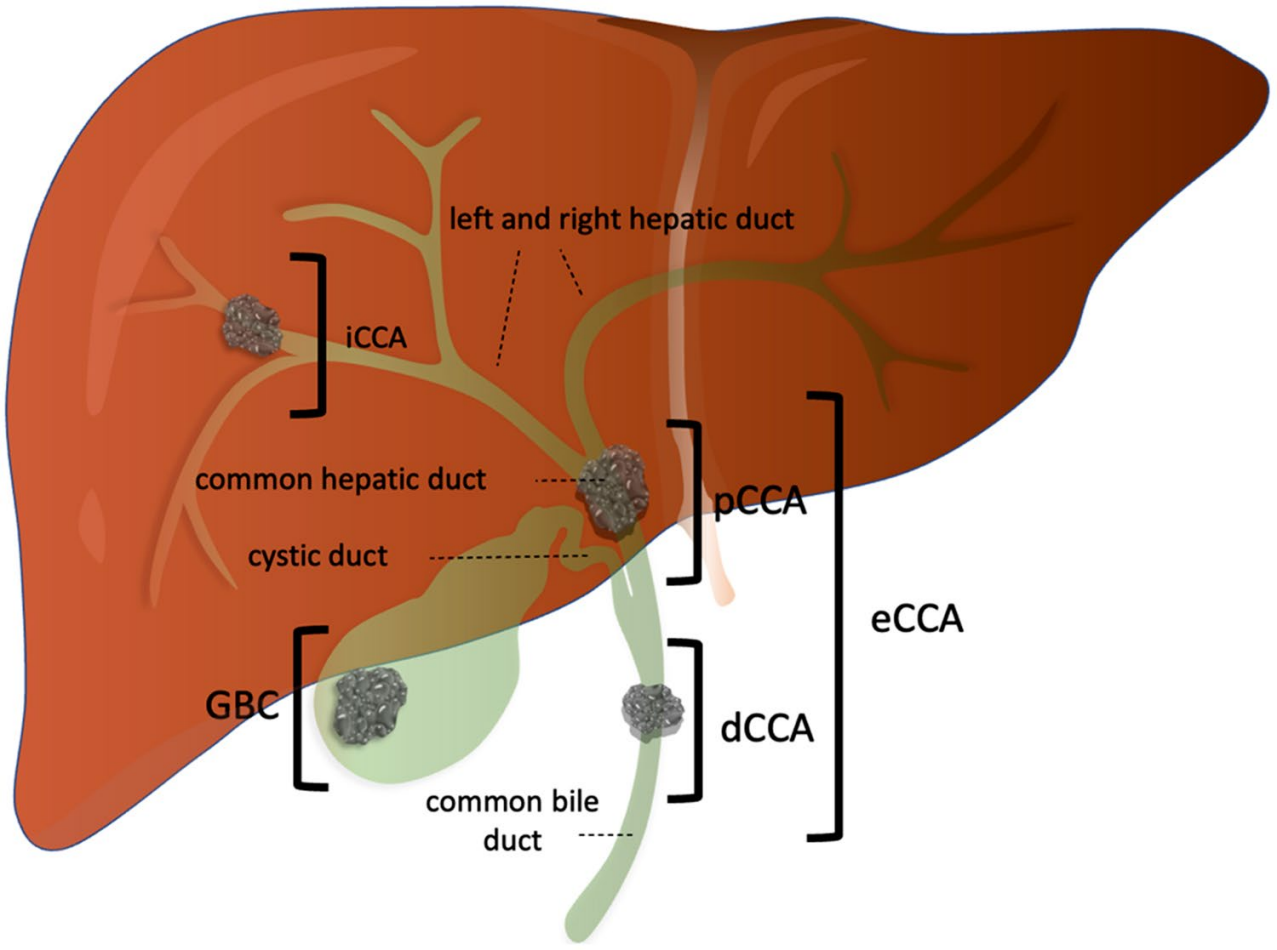
Schematic representation of the anatomic subgroups of biliary tract cancer. The classification of the carcinomas is based on the anatomical localization of the primary tumor. Intrahepatic cholangiocarinomas (iCCA) originate from the intrahepatic bile tracts proximal to the left or right hepatic duct. Extrahepatic tumors (eCCA) are divided into perihilar (pCCA) and distal (dCCA) carcinoma. pCCA—also called Klatskin tumors—compromise tumors of the left and right hepatic ducts and the common hepatic duct. dCCA are localized distal to the estuary of the common hepatic duct into the common bile duct. Carcinoma of the gallbladder (GBC) and of the cystic duct are subentities

Cholangiocarcinoma (CCA) is the second most common group of primary liver malignancies[1]. Depending on the localization of the primary tumor, it is divided into intrahepatic (iCCA) and extrahepatic (eCCA) tumors, which are further divided into distal and perihilar (so-called Klatskin) carcinomas. A special form are carcinomas of the gallbladder, which are commonly subsumed under biliary tract cancer in clinical studies[2] (Fig. 1).

Due to different prevalences for risk factors, the incidences of the different tumor localizations of CCA vary considerably worldwide[3]. In Germany, the overall incidence has increased within the last 20 years and currently stands at more than 7000 new cases per year[1].

In addition to the growing incidence of tumors, the available treatment options are increasing, which is also reflected in the current international recommendations for the treatment of CCA[4–8]. The new edition of the German S3 guideline "Diagnostics and Therapy of Hepatocellular Carcinoma and Biliary Carcinomas", published in June 2021, is—for the first time—also dedicated to the diagnosis and treatment of bile duct carcinomas. Among other things, the increasing importance of locoregional forms of therapy is discussed here[1, 9].

Radical surgical resection to tumor-free margins is still the only curative therapy for non-metastatic CCA[7]. Particularly in the case of iCCA however, due to long asymptomatic phases which often lead to an advanced tumor stage at initial diagnosis, less than 30–40% of patients are operable [5, 10]. In addition, there is a high risk of recurrence at 40–80% after surgical tumor resection[1].

In the case of locally advanced tumors or in the metastatic state, systemic therapy with gemcitabine and cisplatin is generally recommended as first-line therapy. With this combination therapy, a median overall survival of about 12 months can be achieved[11]. As an alternative or as a supplement to systemic therapy, various local intra-arterial therapies are available, in particular in patients with a liver-dominant disease. In addition to transarterial chemoembolization (TACE), transarterial radioembolization (TARE) and hepatic arterial infusion (HAI), the S3 guideline also lists chemosaturation with percutaneous hepatic perfusion (PHP)[1].

The local therapies can be used not only for local tumor control, but also for down-staging with the aim of secondary resection[1]. The combination of systemic and local therapy has also proven successful in this context in several studies[12, 13]. PHP currently offers the possibility of a palliative life extension as part of an individual therapy plan[14].

PHP is a locoregional form of therapy for the treatment of inoperable primary and secondary liver malignancies. PHP enables high-dose chemotherapy of the liver with reduced systemic exposure and thus fewer systemic side effects.

While there is reliable data on PHP in metastatic uveal melanoma[15–17], studies on the effectiveness of PHP in other solid tumors such as CCA are very limited. Since there are currently no results from prospective studies on PHP in CCA, there is an urgent need for data from *real-world* studies to better classify the effectiveness and safety of PHP in this rare tumor entity.

The aim of this retrospective unicentric study was an analysis of PHP as a palliative therapy plan in patients with inoperable iCCA and CCA liver metastases.

## Material and methods

### Study design

This monocentric retrospective study design was approved by the local ethics committee. All patients with iCCA and CCA liver metastases treated with PHP between April 2014 and September 2020 were included in the analysis. In all patients, PHP was regarded as the most appropriate therapy for the salvage setting after discussion within a multi-disciplinary local tumor board. Criteria for PHP were a liver-dominant tumor distribution and an adequate hematologic, renal, and hepatic function. Contraindications included heart failure with a left-ventricular ejection fraction < 40%, significant chronic obstructive or restrictive pulmonary disorder, intracranial lesions with a high bleeding risk and a history of transient ischemic attacks or stroke within the last six months[14, 18].

#### Chemosaturation with percutaneous hepatic perfusion

The PHP procedure has already been described in detail in various studies[14, 15, 18–22]. A catheter, inserted via a sheath in the left common femoral artery and positioned in the tumor-supplying liver artery, is used for chemoperfusion with melphalan (2.5–3 mg/kg ideal body weight with a max. dose of 220 mg). Melphalan—which is not commonly used in CCA—is the only agent approved for the PHP procedure.

**Fig. 2:**
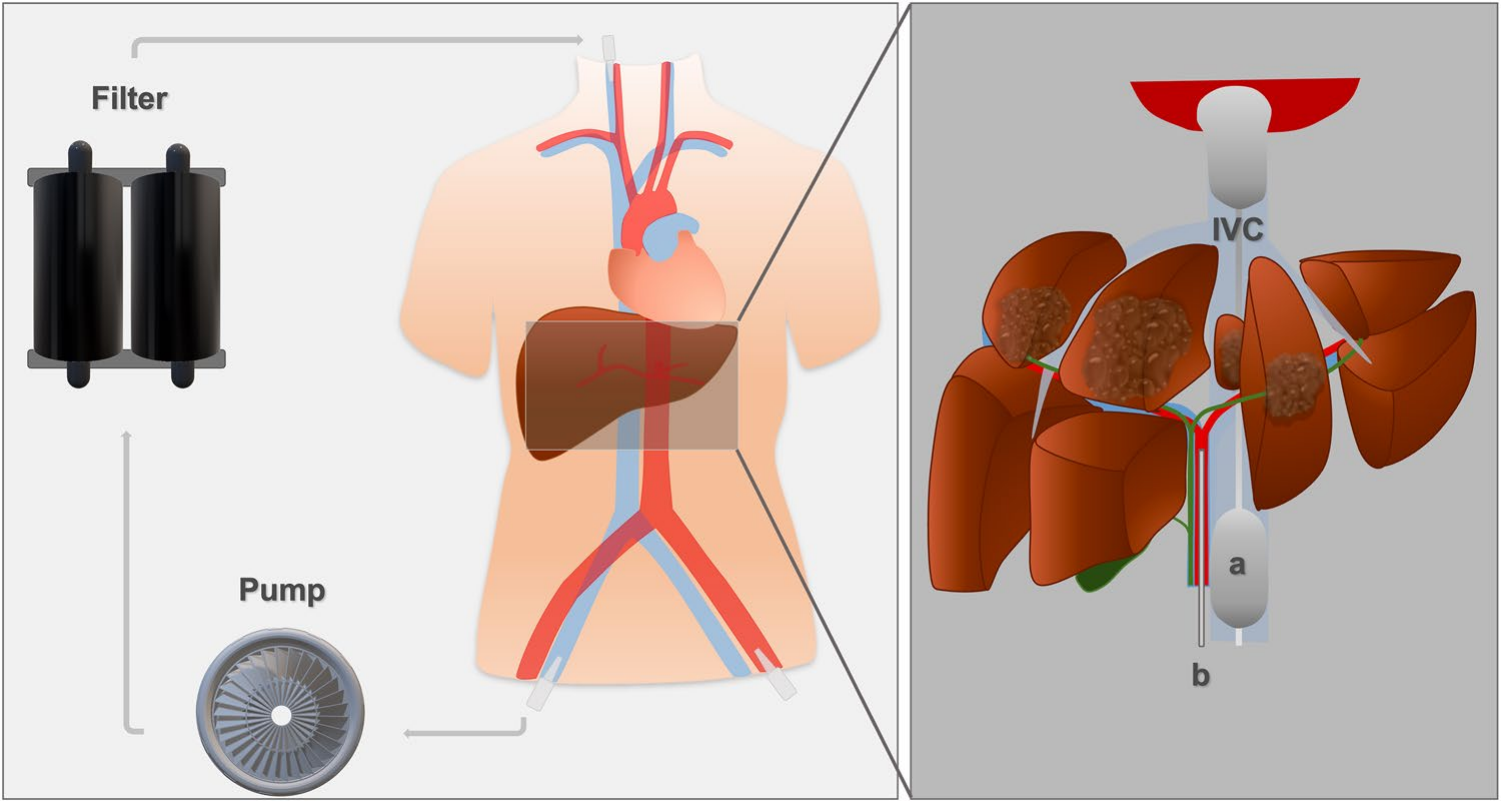
Schematic overview of the percutaneous hepatic perfusion (PHP) setup. The inferior vena cava (IVC) is isolated from the systemic circulation using the balloons of the double balloon catheter (**a**). High-dose chemotherapy (melphalan) is administered via a (micro-)catheter positioned in the tumor-supplying liver artery (**b**). The venous hepatic blood is aspirated through the double balloon catheter and sucked into an extracorporeal circuit by a pump. Following, an extracorporeal filter cleanses the melphalan-enriched blood before returning it to the systemic circulation via a sheath in the right jugular vein

Via the right common femoral vein, a 16 French double balloon aspiration catheter is placed in the inferior vena cava (IVC). By inflation of the balloons, the hepatic segment of the IVC is isolated (Fig. 2). The melphalan-enriched blood is aspirated and filtered in an extracorporeal melphalan-specific filtration system (with an extraction rate of ca. 96%). Following, the cleansed blood is returned to the circulation via a sheath in the right jugular vein. To prevent clotting within the extracorporeal system, unfractionated heparin is injected; an activated clotting time (ACT) above 500 s is mandatory.

Due to the lengths of the intervention and the frequent fluctuations in blood pressure and heart rate during the initiation of the extracorporeal circuit, all PHP are performed under general anesthesia[23].

After the first PHP treatment, subsequent PHP were optional for patients with stable disease (SD), partial response (PR) or complete response (CR) according to the Response Evaluation Criteria In Solid Tumors 1.1(RECIST)[24] in the first radiological control. Contraindications for further PHP treatment were progressive disease (PD) according to RECIST 1.1 or poor tolerance of the first PHP.

#### Analysis of toxic side effects and therapy-associated complications

Peri-interventional complications and toxicity were classified using the Common Terminology Criteria for Adverse Events (CTCAE v5.0), which divides adverse events (AEs) into five grades (mild; moderate; severe, life-threatening; deadly). Basis for this were clinical reports and hematologic, hepatologic and biliary laboratory values assessed pre-interventional (considered as baseline values) and on day 1,3,7,14 and 21. The peri-interventional mortality was computed.

#### Analysis of treatment response

The first radiologic follow-up exam (CT or MRI) was conducted median 6.4 (interquartile range (IQR) 5.8–8.9) weeks after the PHP. RECIST 1.1 was used to assess tumor response. Disease control rate (DCR) was defined as PR, CR or SD according to RECIST 1.1. The overall response rate (ORR) was defined as PR or CR. The median time from the first PHP to overall response was defined as time to response (TTR).

#### Survival analysis

The median overall survival (mOS) was calculated from first diagnosis of the CCA and from first PHP treatment to the last follow-up exam or date of death. The median progression-free survival (mPFS) was computed from first PHP to radiological tumor progress, last follow-up or death (whichever occurred first). The median *hepatic* progression-free survival (mhPFS) was assessed in the same manner.

#### Statistical analysis

Data were retrospectively extracted from digital patient records. Survival curves were calculated with Kaplan–Meier-Estimator (using GraphPad Prism version 9.2.0). Logrank (Mantel-Cox) test was used to compare survival curves. Continuous data were tested using Mann–Whitney U calculations. A p-value of < 0.05 was determined to be significant.

Parts (*n* = 15) of the patient cohort were already reported on, with shorter observational periods and less PHP procedures however [10, 14].

## Results

Patient characteristics and procedural data.

In total 17 patients with histologically proven iCCA or CCA liver metastases were treated with 42 PHP. Thus, the patients were treated with 2.5 PHP on average (min. 1; max. 8). Among the 17 patients were 13 patients with iCCA. Three patients with distal eCCA were initially treated with biliary resection and biliodigestive anastomosis and developed intrahepatic metastases in the course of their disease. One patient with Klatskin tumor was initially treated with extended hemihepatectomy, segmental resection of the portal vein, biliary resection and biliodigestive anastomosis.

The PHP procedures proceeded without CTCAE grade 3–5 complications. Hypotension and tachycardia, which were proficiently treated with volume expansion and catecholamine substitution, occurred during all procedures.

A detailed overview of the patient characteristics and procedural parameters are provided in Table 1.

**Table 1:**
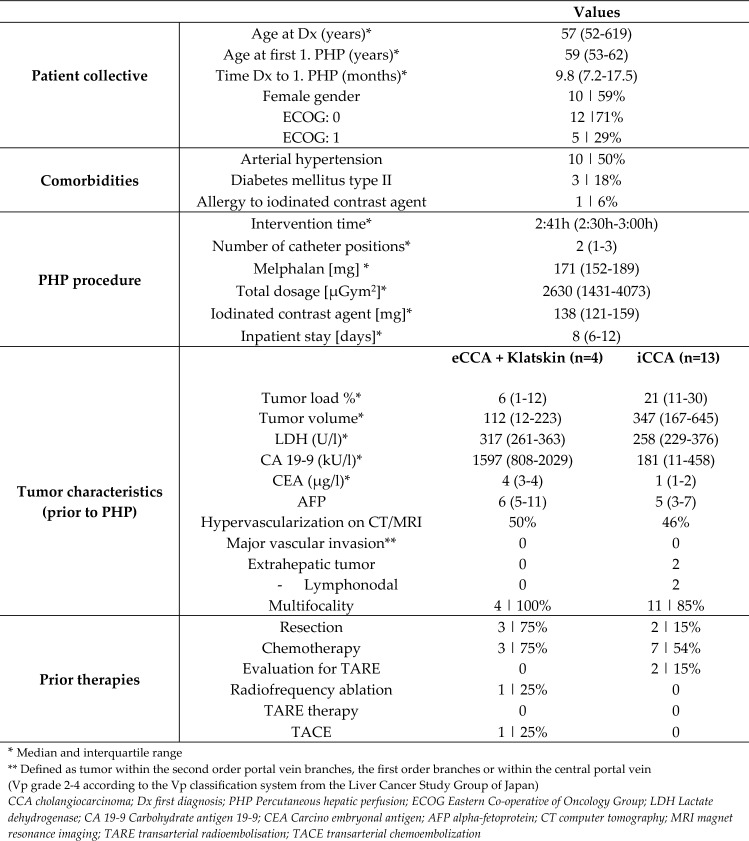
Overview of interventional parameters and characteristics of 17 patients with iCCA, Klatskin tumor or eCCA liver metastases

## Response to therapy

**Fig. 3:**
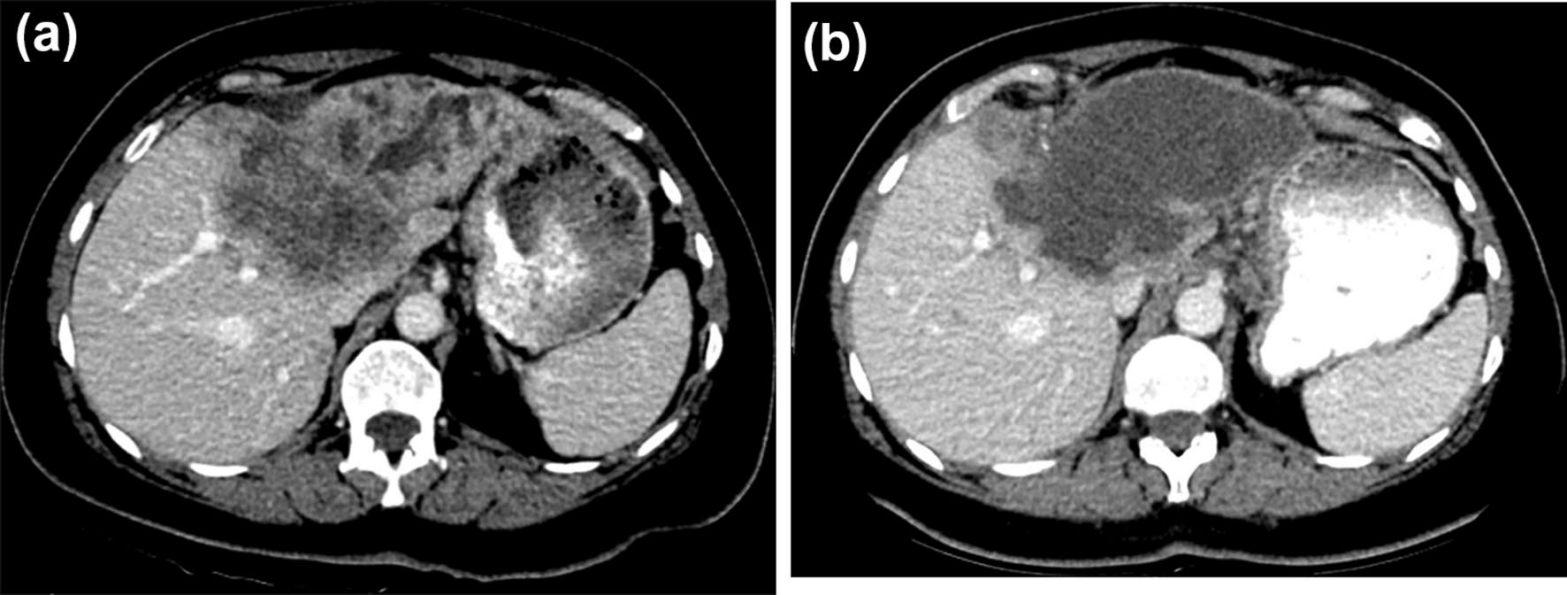
Computed tomography images of a patient with intrahepatic cholangiocarcinoma. At first diagnosis, the tumor is unresectable. Several systemic chemotherapies with gemcitabine/ cisplatin and liposomal irinotecan/ 5-fluorouracil/ folinic acid are without success. After exhaustion of the standard therapies the interdisciplinary tumor board suggests a chemosaturation with percutaneous hepatic perfusion. **a** Shows the pre-interventional tumor: a large mass with multiple small satellite masses is located on the border of the liver segments V/VIII. Cholestasis is present in the left liver lobe. **b** In the first follow-up imaging 7 weeks after the first PHP, large areas if the tumor are necrotic

One patient deceased without follow-up imaging 13 weeks after the first PHP with no identifiable link to the PHP treatment. Thus, response data of 16 patients are available. After the first PHP, one patient presented with complete response (CR: 6%). Three patients showed a partial response in the first follow-up exam (PR: 18%) and seven patients presented with a stable disease (SD: 44%). Figure 3 depicts an example of a patient with a promising therapy response. Five patients had a tumor progression (PD: 31%), one of which was limited to extrahepatic progression only (pulmonary metastases).

Two patients with PR, six patients with SD and the patient with PD limited to extrahepatic progression received further PHP treatments. In the subsequent follow-up exams, the overall best therapy response in these patients was PR in 78% and SD in 22%. One patient was treated in total with 8 PHP within 30 months. In the follow-up exams an efficient response to the treated tumors was noticeable, but also new tumor lesions after the 1st, 2nd and 4th to 8th PHP (formal PD), which could be addressed with other locoregional therapies (radiosurgery; radio frequency ablation; microwave ablation; resection). Further on, when the tumor had recurred, it was re-treated with PHP. Of note, the PHP treatment was unrelated in time to the other locoregional therapies, it was never used as combination therapy. For the response evaluation, we exclusively used follow-up exams directly subsequent to the PHP treatment.

In total, in 17 treated patients an ORR of 25% and a DCR of 75% was achieved. The TTR was 44 days.

## Survival

**Fig. 4:**
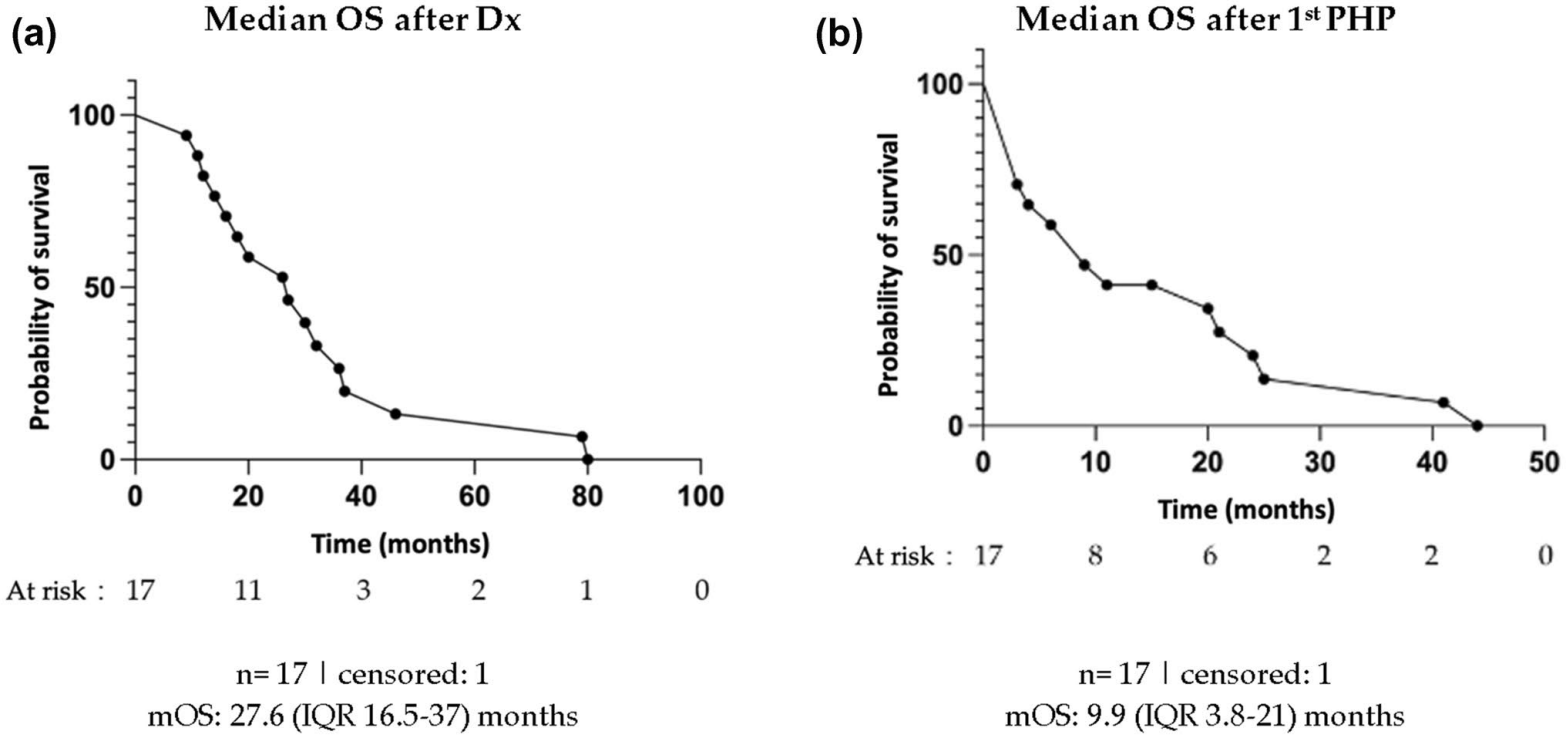
Survival analysis of 17 patients receiving chemosaturation with percutaneous hepatic perfusion (PHP): median overall survival (OS) after first diagnosis (Dx) of intrahepatic cholangiocarcinoma or intrahepatic metastases of CCA (**a**); median OS after the first PHP (**b**). *IQR interquartile range*

The median PFS was 3.5 (2.2–7.4) months and similar to the median hepatic PFS (3.6 (2.6–9.5) months; *p* = 0.79). Calculated from first diagnosis of iCCA (or CCA liver metastases), the median survival was 27.6 (16.5–37) months. From first PHP, a median survival of 9.9 (3.8–21) months was observed, with a 1-year survival rate of 41% (see Fig. 4).

## Complications

The day following the fifth PHP one patient developed a transient hemiparesis. Cerebral MRI revealed an ischemic (most likely thromboembolic) insult in the left precentral cortex. No abnormalities were found on further neurologic and cardiologic tests. Lysis was not performed due to the mild symptoms, which resolved spontaneously within hours. Post-interventional CTCAE grade 3–4 were rare; one patient developed an aspiration pneumonia as a consequence of postoperative nausea and vomiting (PONV), which regressed under antibiotic treatment. Another patient suffered from a persistent catecholamine-dependent hypotension and thus remained on intensive care unit (ICU) for 72 h. No PHP-related deaths occurred (peri-interventional mortality: 0%).

## Toxicity

**Fig. 5:**
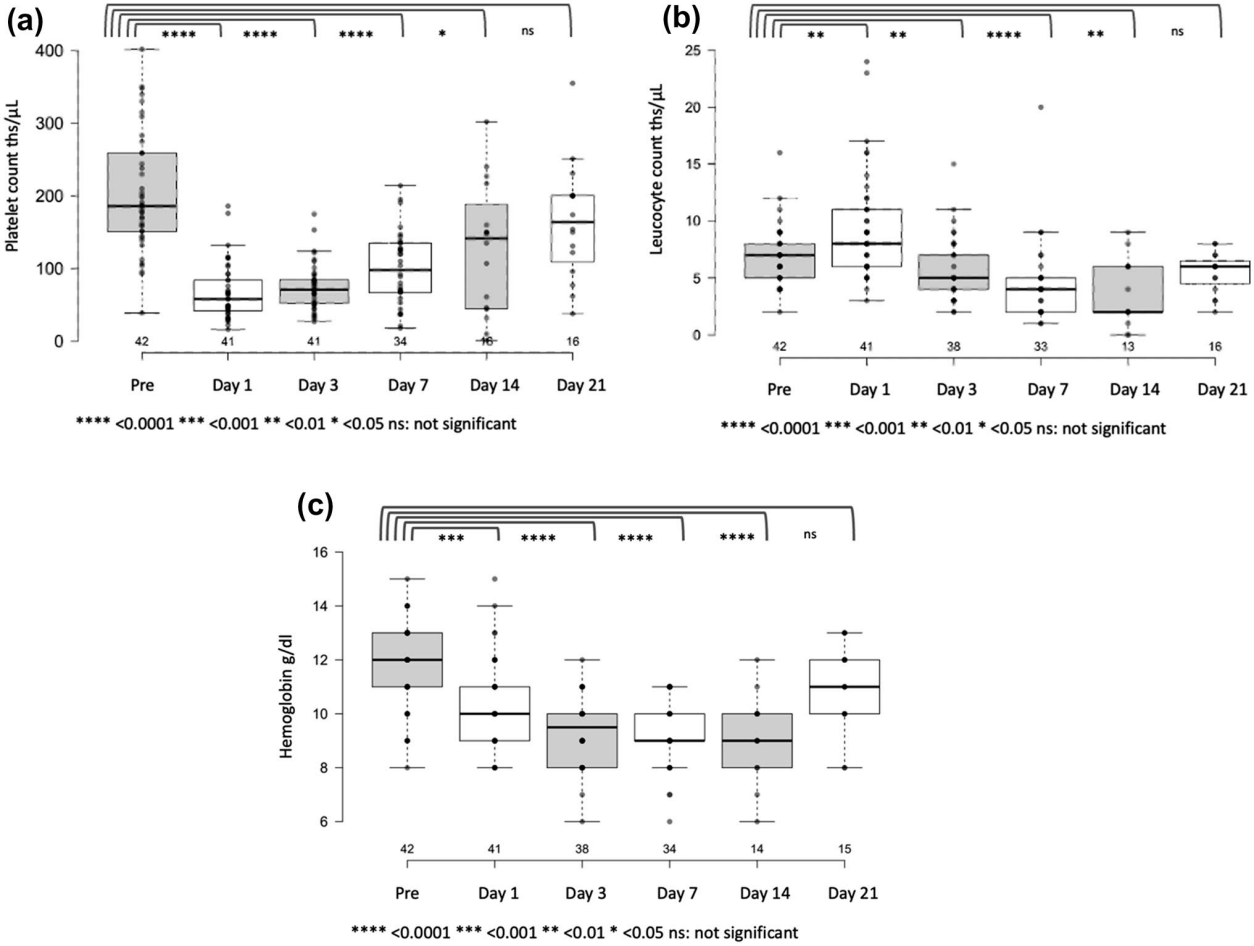
Time line of platelet count (**a**), leucocyte count (**b**) and hemoglobin values (**c**) before (pre) and up to 21 days after chemosaturation with percutaneous hepatic perfusion (PHP). Die black line marks the median value. The different assessment times were tested for significance using the Mann–Whitney U test

After in total 50% of the PHP procedures clinically significant thrombocytopenia (CTCAE grade 3 or 4) developed. The second most common hematotoxic side effect was grade 3/4 anemia (after 26% of the PHP), followed by leucopenia after 21% of the interventions (Table 2). This myelosuppressive effect was transitory and the laboratory values returned to baseline within 3 weeks (Fig. 5). Hepatotoxic toxicity commonly resulted in an increase of liver enzymes (AST increase after 21% and ALT increase after 24% of the PHP), but rarely manifested as a restricted liver synthesis capacity (hyperbilirubinemia after 5% and hypalbuminemia after 14% of the PHP). A combined increase in liver enzymes and decrease in synthesis capacity was not observed.

**Table 2:** Hematologic, hepatic, and biliary adverse events grade 3 and 4 (classified by CTCAE v5.0) of patients after the first and after any PHP

	AEs after 1st PHP *n* = 17	AEs after any PHP *n* = 42
**Thrombocytopenia**		
Grade 3	5 | 29%	16 | 38%
Grade 4	2 | 12%	5 | 12%
*Grades 3* + *4*	*7 | 41%*	*21 |50%*
**Leucopenia**		
Grade 3	2 | 12%	6 | 14%
Grade 4	1 | 6%	3 | 7%
*Grades 3* + *4*	*3 |18%*	*9 |21%*
**Anemia**		
Grade 3	5 | 29%	11 | 26%
Grade 4	0	0
*Grades 3* + *4*	*5 | 29%*	*11 | 26%*
**AST increase**		
Grade 3	4 | 24%	9 |21%
Grade 4	0	0
*Grades 3* + *4*	*4 | 24%*	*9 |21%*
**ALT increase**		
Grade 3 Grade 4	1 | 6%	4 | 10%
	1 | 6%	2 | 5%
*Grades 3* + *4*	*2 | 12%*	*6 | 14%*
**Hyperbilirubinemia**		
Grade 3	0	2 | 5%
Grade 4	0	0
*Grades 3* + *4*	*0*	*2 | 5%*
**Hypoalbuminemia**		
Grade 3	2 | 12%	6 | 14%
Grade 4	0	0
*Grades 3* + *4*	*2 | 12%*	*6 | 14%*

*AE* adverse event, *PHP* percutaneous hepatic perfusion, *CTCA E* Common Terminology Criteria for Adverse Events, *AST* aspartate aminotransferase, *ALT* alanine transaminase

## Discussion

The results of this monocentric retrospective study show that chemosaturation with percutaneous hepatic perfusion (PHP) with melphalan is an effective and safe treatment option for patients with advanced cholangiocarcinoma (CCA).

The only curative treatment for iCCA and CCA liver metastases is surgical resection, but this is no longer possible in the majority of patients at diagnosis. In the inoperable state, the treatment options for biliary tract cancer are limited and the prognosis is poor: untreated, a median survival of 2.5–6 months is to be expected [8], which can be extended to approx. 12 months under the first-line chemotherapy with gemcitabine and cisplatin[1, 11, 25].

Locoregional therapies play an increasingly important role not only in palliative approaches, but also in multimodal combined or sequential therapies and even in the neoadjuvant treatment of CCA: Locally ablative methods such as radiofrequency ablation (RFA) and microwave ablation (MWA) are established both in the first-line therapy of primary CCA up to 3 cm in size and in the treatment of recurrent tumors[1]. Analogous to hepatocellular carcinoma (HCC), a combination therapy of selective chemo-embolization and ablation can also be carried out in somewhat larger tumors[1, 9].

Intraarterial procedures (e.g. TARE, TACE, HAI and PHP) are mainly used in patients whose tumors cannot be treated focally (by resection or ablation) due to size, extent or localization. The administration of therapeutic agents directly into the tumor-supplying hepatic artery ensures supra-selective therapy. Nevertheless, both in the case of TACE and in particular in the case of HAI, a spread of the high-dose substances in the systemic circulation can be problematic. In PHP, however, the systemic toxicity of chemotherapeutic agents is efficiently reduced by extracorporeal filtration.

Which intra-arterial therapy approach in which sequence can best be used in which patient group has so far been poorly characterized. The limited value definition of the different therapy approaches is retraceable to a lack of randomized controlled studies. Even if systematic reviews and pooled analyses aim for an approximation, they are characterized by the heterogeneity of the included studies, e.g., with regard to patient collectives, methodology and data acquisition[26].

Overall, our patients achieved a median OS of 9.9 months after starting PHP therapy. Taking into account the prior therapies (all patients were previously treated at least with systemic chemotherapy), this result confirms the potential for a life extension by the PHP even after exhaustion of the systemic therapies. Based on the initial diagnosis, the median survival was 27.6 months. Interpretation of these results is hampered by the limited data available on the efficiency of PHP in CCA[27]. In 2019, we were able to determine a median OS after PHP of 7.6 months and a median OS after initial diagnosis of 26.9 months in a multicenter evaluation of 15 iCCA patients from nine centers, as well as a local tumor control of 53%[10]. In a prospective study, eleven patients with hepatobiliary tumors were evaluated, but no distinction was made between hepatocellular and cholangiocellular carcinomas [28].

Similar to our analysis, the previously published work on other intra-arterial therapy procedures (TACE, TARE and HAI) also shows a wide range with regard to survival times in CCA. A large US study of TACE examined mostly pretreated patients and showed a median OS of 15 months after the start of TACE therapy and of 20 months after initial diagnosis[26, 29]. A meta-analysis by Ray et al. in turn looked at 16 studies (*n* = 542 patients) and determined an OS of 13.4 months after the first TACE and 15.7 months after initial diagnosis [30]. With regard to TARE, Cucchetti et al. investigated a total of 224 patients in a meta-regression study and calculated an OS of 11.5 months from the start of TARE therapy in pretreated patients and an OS of 24 months in patients with TARE as first-line therapy[31].

In 2015, Boehm et al. calculated a median OS of 22.8 months after the first HAI in a meta-analysis of 4 studies/ 79 patients[32]. While this survival rate of HAI appears to be superior to that of TARE, TACE and PHP, there was also a higher rate of Grade 3 and 4 adverse events (according to WHO or NCI CTCAE) compared to TACE and TARE (HAI: 0.35; TACE: 0.26; TARE: 0.32)[32]. In addition, there is an increased risk of complications in HAI due to the necessity of implanting a port system or another catheter system. Possibly because of the comparatively higher complication rate, the HAI is not recommended in the guideline of the International Liver Cancer Association (ILCA) (recommendation category C2)[8].

Melphalan, which is an alkylating agent that is not cell-cycle-specific[33], is not commonly used in the treatment of CCA. This might lead to the question, where it`s effect in PHP comes from. The principle of PHP allows an extreme dose-escalation of melphalan within the liver while limiting the systemic exposure due to the extracorporeal filtration. Most likely, the tumor-related toxicity in PHP is generated by the explicitly high dose of chemotherapy delivered to the tumor.

The significant hematotoxic and hepatotoxic side effects in our study were clinically controllable and transient overall. Thrombopenia occurred most frequently (after 50%), followed by leukopenia (after 26% of the interventions). These toxicity rates are consistent both with previously published PHP values[10, 14, 16–18, 21], and with the results of the ABC-02 study[34], which set a milestone for first-line system therapy with gemcitabine/cisplatin in CCA.

Combination therapies are also increasingly being investigated in the treatment of CCA. This follows a general trend in interventional oncology, which evaluates the synergistic effects of local therapies and systemic therapies. Two recent phase II studies on combination therapy with gemcitabine/platinum derivatives plus TARE (as first-line therapy) [13] or HAI (as first-line therapy in 92% of patients)[12] present promising survival times (TARE mOS: 22 months; HAI mOS: 25 months). The order and sequence in which local and systemic therapies are best combined has not yet been conclusively clarified. However, a randomized controlled phase III study of response and survival after PHP plus systemic therapy with cisplatin/gemcitabine versus systemic therapy alone with gemcitabine/cisplatin is currently in progress (ClinicalTrials.gov Identifier: NCT03086993).

In addition to classical chemotherapy, targeted (in IDH-1 mutated tumors) and immunomodulating therapies (e.g. with isocitrate dehydrogenase 2 or fibroblast growth factor receptor inhibitors) are promising substances classes for potential (combination) therapies, as they could not only perspectively provide a further individualization of the treatment, but also as they open up possibilities for new combinations with local therapies.

Our study has significant limitations. Due to the retrospective study design, adverse events may have been underestimated, especially since some patients were partially assigned from a distance and monitored close to home. Therefore, follow-up laboratory values are not available for all patients at the specified times. There are a number of factors influencing the post-interventional course: despite the standard operation procedure, medical follow-up can be subject to individual fluctuations and thus affect the side effect profile. Moreover, preceding therapies and their effects on survival times must be considered. Furthermore, the limited comparability to other studies regarding response rates and, in particular, survival rates due to the different patient population should also be emphasized.

Overall, this study shows that the palliative concept of PHP has the potential to prolong life in patients with inoperable, treatment-refractory iCCA and CCA liver metastases. In order to assess which intra-arterial therapy or combination therapy is advantageous for which patient group, further, preferably prospectively randomized studies with larger numbers of patients, are necessary.

## Data Availability

The data presented in this study are available on request from the corresponding author. The data are not publicly available due to privacy.
